# Elastic solution of surface loaded layer with couple and surface stress effects

**DOI:** 10.1038/s41598-023-27705-1

**Published:** 2023-01-19

**Authors:** Jintara Lawongkerd, Toan Minh Le, Wipavee Wongviboonsin, Suraparb Keawsawasvong, Suchart Limkatanyu, Chung Nguyen Van, Jaroon Rungamornrat

**Affiliations:** 1 Department of Civil Engineering, Faculty of Engineering, Thammasat School of Engineering, Thammasat University, Pathumthani, 12120 Thailand; 2 Center of Excellence in Applied Mechanics and Structures, Department of Civil Engineering, Faculty of Engineering, Chulalongkorn University, Bangkok, 10330 Thailand; 3 Department of Civil and Environmental Engineering, Faculty of Engineering, Prince of Songkla University, Songkhla, 90112 Thailand; 4 Faculty of Civil Engineering, Ho Chi Minh City University of Technology and Education, Ho Chi Minh City, 721400 Vietnam

**Keywords:** Engineering, Mathematics and computing, Nanoscience and technology

## Abstract

In this study, an elastic solution of an axisymmetrically surface-loaded thin layer resting on a rigid substrate is established by taking the surface stress and material microstructural effects into account. Derived solutions provide not only a means to investigate the size effects on the mechanical response but also a set of fundamental solutions essential for tackling contact problems in a micro/nano scale. In the formulation, the couple stress and surface elasticity theories are adopted to simulate the microstructured bulk layer and the surface material, respectively. A general solution of an elastic field within the bulk layer is obtained first by Hankel transform method and subsequently used together with the surface equations and boundary conditions to form a set of conditions essential for determining all unknown constants. After being fully tested with available benchmark solutions, results are used to study the role of surface and couple stresses on the load transferring mechanism to the substrate and its size-dependent characteristic for a wide range of external length scales relative to the internal length scales.

## Introduction

Coatings to enhance the surface and overall properties of objects have been found in various disciplines including food science (e.g., food packaging, kitchen tools, and counter-tops kill bacteria/microbes, etc.), building constructions (e.g., interior and exterior house paints, interior furnishings, glass and facade coatings for high-rise buildings, etc.), costumes (e.g., stain-proof clothing, protection suit, etc.), vehicles and structures (e.g., spacecraft, airplanes, automobiles, bridges, road markings, marine vessels, etc.), a wide variety of industrial and non-industrial maintenance coatings, and numerous electronic and biomedical products. In recent years, applications of nanotechnology to enhance performance of surface coatings have grown remarkably. Such continuous developments and uses of nanoscale coatings result directly from the increasing availability of nanoscale/nanostructured materials and advances in the coating processes. For instance, silver nanoparticles embedded in textiles can kill odor-causing bacteria; nanofiber coatings on textiles can stop liquid penetration; novel nanomaterials on fabrics can also absorb perspiration and wick it away; and titanium nanoparticles embedded in textiles can inhibit UV rays from penetrating through the fabric, etc^1^.

Many researches have been extensively conducted to understand the fundamental behavior of micro- and nano-structures such as micro-/nano-scale beams^2,3^, plates^4,5^, surface coating^6–8^ and indentations^9,10^. Most of the existing studies can be divided into three main groups based on the underlying methodology and procedure employed: one associated with experimental investigations^11–13^ and the other two concerning discrete-based^14–18^ and continuum-based mathematical modelings. In the past decades, simulations based on continuum-based mathematical models have been progressively offered as viable alternatives. Various size-dependent elasticity theories, such as the couple stress theory^19–23^, the strain-gradient-based elasticity theory^24,25^, the surface stress elasticity theory^26–28^, and the nonlocal elasticity theory^29–31^, have been proposed to account for the influence of material small-scale structures in a continuum manner. Although the results and findings from mathematical models are considered only as of the first/rough response estimation, these predicted trends can be used to provide preliminary data for more accurate experiments.

Fundamental problems in solid mechanics at micro/nano scales are extensively studied, especially those involving surface loads and contacts. Several groups of researchers have studied the size-dependent effects using various theories. Couple-stress-based theories, in which an additional deformation measure termed the curvature is introduced along with its conjugate pair known as the couple stresses, are commonly used in the literature to simulate the influence of material microstructures of small-scale objects. The original (indeterminate) couple stress theory was proposed by Mindlin and Tiersten^19^, Toupin^20,21^, Mindlin^22^, and Koiter^23^ and has been received attention from researchers due to its capability to tackle problems at micro-scale. Muki and Sternberg^6^ first applied the theory to investigate the role of couple stresses on the response of an elastic half plane under surface loads and simple contacts. Since then, studies have been significantly expanded to handle more complex scenarios including indentation problems^32–37^ and layered media^38–42^. The nontrivial extension to three-dimensional cases has also been documented^43–46^. Nevertheless, the number of studies is still relatively few in comparison with that of two-dimensional problems.

The surface/interface elasticity theory is one among available frameworks widely adopted to simulate the mechanical response of tiny-scale objects in which the surface free energy is observed to be significant. The solid mathematical foundation of such theory was laid down by Gurtin and his co-workers^26–28^ by following the fundamental idea of Gibbs^47^ and its modeling capability in comparison with atomistic and molecular static simulations has been confirmed by several studies^48–50^. Within the context of surface mechanics, applications of such theory to study the near-surface responses have also been well recognized; for instance, problems related to half plane, half space, and layered media under surface loads (e.g.^7,8,51–54^) and surface contacts^55–62^. Results from existing studies have confirmed the significant role of both residual surface tension and surface elasticity on the predicted responses and the size dependent characteristics as the relevant external length scales become comparable to the intrinsic length scale of the material surface. In such tiny scales, the need to replace the conventional, size-independent mechanics theory with models capable of accounting for size effects is apparent.

While both microstructure of bulk materials and surface free energy have been addressed to be responsible for the size-dependent characteristics of the response of micro/nano-scale homogeneous and layered media, work toward the integration of both effects in the simulations, within the continuum-based framework, has been still relatively few. Recently, Le et al.^63^ and Le et al.^64^ applied both couple stress and surface elasticity theories to investigate the size dependent response of a homogeneous half plane excited by surface loads and tilted flat indenters, respectively. The extension to treat a surface loaded homogeneous half space accounting for both couple and surface stresses was achieved by Lawongkerd et al.^65^. In above mentioned studies, it was clearly demonstrated that both effects are significant when the internal length scales of the bulk and surface materials are comparable. The simultaneous effects must be taken into account in the modeling when the relevant external length scales fall within the range of the two material length scales. While the role of both surface and couple stresses were extensively explored in above investigations, the medium was modeled either by a homogeneous half-plane or half-space, and such simplified settings clearly pose a key limitation on their practical applications. For instance, key response and characteristics of coated objects with a very thin coating layer under surface excitations (e.g., load transferring mechanism to coated substrate and influence of coating-layer thickness) is not possible from such limited settings. On the basis of an extensive literature survey, the authours are unaware of any further development from the studies mentioned.

In the present study, a size-dependent elastic response of a surface loaded material layer resting on a substrate is investigated. Both surface free energy and bulk material microstructures are taken into account in the modeling as those responsible for size effects. The treatment of a medium as a finite-thickness layer clearly broadens its practical applications from available half-plane and half-space cases, especially for studying surface coating problems. Besides their direct contribution to gain an in-depth understanding of the mechanical response of a very thin layer medium, established results form an essential and sufficient basis for the development of a solution scheme to tackle surface contact problems.

## Problem formulation

**Figure 1:**
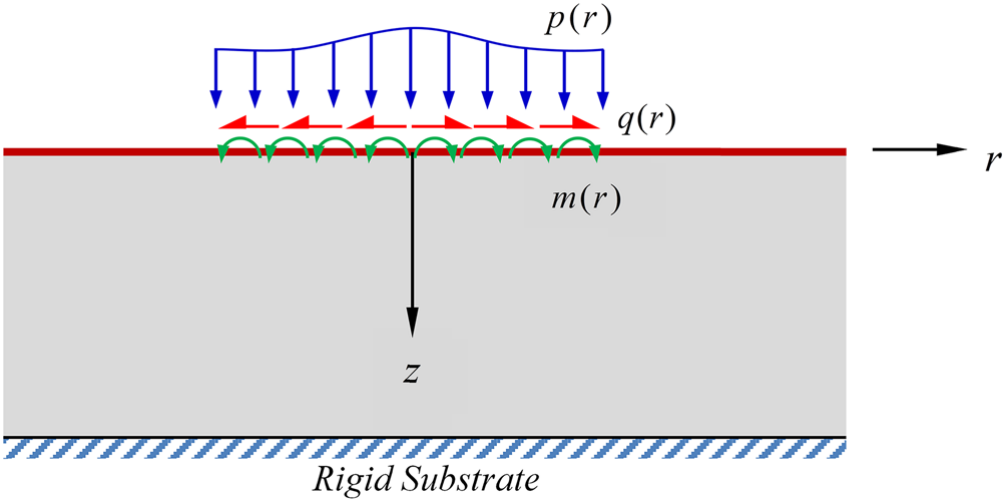
Schematic of a three-dimension, rigid-based, elastic layer and subjected to arbitrarily distributed axisymmetric surface loads.

Consider a three-dimensional, elastic layer of finite thickness *h* (representing a thin coating layer) resting on a rigid substrate (representing a coated substrate) as shown schematically in Fig. 1. The layer consists of a bulk part, which is made of a homogeneous, isotropic, linearly elastic material possessing microstructures, and a surface part, which is perfectly adhered to the top of the bulk and has its own properties. The layer is loaded on the top surface by axisymmetrically distributed normal traction $p$, shear traction $q$, and couple traction $m$ over a circular region of radius $a$ and free of traction elsewhere. In the formulation and solution scheme presented further below, a reference cylindrical coordinate system $\{ O;r,\theta ,z\}$ with the origin *O* located at the center of the loading region, the *r-*axis directing along the infinite direction of the layer, and the *z-*axis directing downward is employed.

### Field equations for bulk part

To simulate the elastic response of the bulk material with microstructures, a fundamental couple stress theory proposed by Mindlin and Tiersten^19^ and Koiter^23^ is adopted. Basic equations (i.e., equilibrium equations, constitutive laws, and kinematics) governing the elastic field under the axisymmetric deformation and zero body force and couple are given by^66,67^

1 $$\begin{gathered} \frac{{\partial \sigma_{rr} }}{\partial r} + \frac{{\partial \sigma_{zr} }}{\partial z} + \frac{{\sigma_{rr} - \sigma_{\theta \theta } }}{r} = 0, \, \frac{{\partial \sigma_{rz} }}{\partial r} + \frac{{\partial \sigma_{zz} }}{\partial z} + \frac{{\sigma_{rz} }}{r} = 0, \, \hfill \\ \frac{{\partial m_{r\theta } }}{\partial r} + \frac{{\partial m_{z\theta } }}{\partial z} + \frac{{m_{r\theta } + m_{\theta r} }}{r} + \sigma_{zr} - \sigma_{rz} = 0 \hfill \\ \end{gathered}$$

2 $$\begin{gathered} \sigma_{rr} = 2\mu \varepsilon_{rr} + \lambda (\varepsilon_{rr} + \varepsilon_{\theta \theta } + \varepsilon_{zz} ), \, \sigma_{\theta \theta } = 2\mu \varepsilon_{\theta \theta } + \lambda (\varepsilon_{rr} + \varepsilon_{\theta \theta } + \varepsilon_{zz} ) \hfill \\ \sigma_{zz} = 2\mu \varepsilon_{zz} + \lambda (\varepsilon_{rr} + \varepsilon_{\theta \theta } + \varepsilon_{zz} ), \, \sigma_{rz} + \sigma_{zr} = 4\mu \varepsilon_{rz} \hfill \\ m_{z\theta } = 4\eta \kappa_{z\theta } , \, m_{\theta z} = 4\eta^{\prime}\kappa_{z\theta } \hfill \\ m_{r\theta } = 4\eta \kappa_{r\theta } + 4\eta^{\prime}\kappa_{\theta r} , \, m_{\theta r} = 4\eta \kappa_{\theta r} + 4\eta^{\prime}\kappa_{r\theta } \hfill \\ \end{gathered}$$

3 $$\begin{gathered} \varepsilon_{rr} = \frac{{\partial u_{r} }}{\partial r}, \, \varepsilon_{\theta \theta } = \frac{{u_{r} }}{r}, \, \varepsilon_{zz} = \frac{{\partial u_{z} }}{\partial z}, \, \varepsilon_{rz} = \varepsilon_{zr} = \frac{1}{2}\left( {\frac{{\partial u_{r} }}{\partial z} + \frac{{\partial u_{z} }}{\partial r}} \right), \, \hfill \\ \kappa_{z\theta } = \frac{{\partial \Omega_{\theta } }}{\partial z}, \, \kappa_{\theta r} = - \frac{{\Omega_{\theta } }}{r}, \, \kappa_{r\theta } = \frac{{\partial \Omega_{\theta } }}{\partial r}, \, \Omega_{\theta } = \frac{1}{2}\left( {\frac{{\partial u_{r} }}{\partial z} - \frac{{\partial u_{z} }}{\partial r}} \right) \hfill \\ \end{gathered}$$

where $\{ \sigma_{rr} ,\sigma_{\theta \theta } ,\sigma_{zz} ,\sigma_{rz} ,\sigma_{zr} \}$ are non-zero force-stress components; $\{ m_{r\theta } ,m_{\theta r} ,m_{z\theta } ,m_{\theta z} \}$ are non-zero couple-stress components; $\{ \varepsilon_{rr} ,\varepsilon_{\theta \theta } ,\varepsilon_{zz} ,\varepsilon_{rz} ,\varepsilon_{zr} \}$ are non-zero components of an infinitesimal strain tensor, $\{ u_{r} ,u_{z} \}$ are non-zero components of the displacement vector; $\Omega_{\theta }$ is a non-zero component of the rotation tensor, $\{ \kappa_{r\theta } ,\kappa_{\theta r} ,\kappa_{z\theta } \}$ are non-zero components of the curvature tensor; $\lambda$ and $\mu$ are Lamé constants defined in the same fashion as that in the classical linear elasticity; and $\eta$ and $\eta^{\prime}$ denote the material constants accounting for the presence of couple stresses. It is worth noting that $\eta$ and $\eta^{\prime}$ are additional material parameters responsible for the length-scale effect (i.e., the presence of material microstructure) and, if these constants vanish, the couple stress theory will reduce identically to the classical linear elasticity.

### Field equations for surface part

A material surface adhered to the top of the bulk is modeled by the surface elasticity theory proposed by Gurtin and Murdoch^26^, Gurtin and Murdoch^27^, and Gurtin et al.^28^. For an axisymmetric case, the non-zero surface displacements $\{ u_{r}^{s} ,u_{z}^{s} \}$, the non-zero surface strains $\{ \varepsilon_{rr}^{s} ,\varepsilon_{\theta \theta }^{s} \}$, and the non-zero surface stresses $\{ \sigma_{rr}^{s} ,\sigma_{\theta \theta }^{s} ,\sigma_{rz}^{s} \}$ are governed by

4 $$\frac{{d\sigma_{rr}^{s} }}{dr} + \frac{{\sigma_{rr}^{s} - \sigma_{\theta \theta }^{s} }}{r} + t_{r}^{s} + q(r) = 0, \, \frac{{d\sigma_{rz}^{s} }}{dr} + \frac{{\sigma_{rz}^{s} }}{r} + t_{z}^{s} + p(r) = 0$$

5 $$\begin{gathered} \sigma_{rr}^{s} = \tau^{s} + (2\mu^{s} + \lambda^{s} )\varepsilon_{rr}^{s} + (\lambda^{s} + \tau^{s} )\varepsilon_{\theta \theta }^{s} , \, \hfill \\ \sigma_{\theta \theta }^{s} = \tau^{s} + (2\mu^{s} + \lambda^{s} )\varepsilon_{\theta \theta }^{s} + (\lambda^{s} + \tau^{s} )\varepsilon_{rr}^{s} , \, \hfill \\ \sigma_{rz}^{s} = \tau^{s} \frac{{du_{z}^{s} }}{dr} \hfill \\ \end{gathered}$$

6 $$\varepsilon_{rr}^{s} = \frac{{du_{r}^{s} }}{dr}, \, \varepsilon_{\theta \theta }^{s} = \frac{{u_{r}^{s} }}{r}$$

where $\tau^{s}$ denotes the residual surface tension; $\lambda^{s} ,\mu^{s}$ are surface Lame’s constants; and $t_{r}^{s} ,t_{z}^{s}$ are radial and vertical traction acting to the surface part by the bulk layer. Combining Eqs. (4)–(6) yields the equilibrium equations in terms of the surface displacements $u_{r}^{s} ,u_{z}^{s}$ as

7 $$\kappa^{s} \left( {\frac{{d^{2} u_{r}^{s} }}{{dr^{2} }} + \frac{1}{r}\frac{{du_{r}^{s} }}{dr} - \frac{{u_{r}^{s} }}{{r^{2} }}} \right) + t_{r}^{s} + q(r) = 0$$

8 $$\tau^{s} \left( {\frac{{d^{2} u_{z}^{s} }}{{dr^{2} }} + \frac{1}{r}\frac{{du_{z}^{s} }}{dr}} \right) + t_{z}^{s} + p(r) = 0$$

where $\kappa^{s} = 2\mu^{s} + \lambda^{s}$ and the fact that the residual surface tension $\tau^{s}$ is spatially independent has been utilized.

### Boundary and continuity conditions

Since the surface part is perfectly bonded to the bulk layer, the surface displacements $\{ u_{r}^{s} ,u_{z}^{s} \}$ and the tractions $\{ t_{r}^{s} ,t_{z}^{s} \}$ can be related to the displacements and stress components of the bulk layer by

9 $$u_{r}^{s} = \left. {u_{r} } \right|_{z = 0} , \, u_{z}^{s} = \left. {u_{z} } \right|_{z = 0}$$

10 $$t_{r}^{s} = \left. {\sigma_{zr} } \right|_{z = 0} , \, \left. {t_{z}^{s} = \sigma_{zz} } \right|_{z = 0}$$

By applying the continuity conditions Eqs. (9) and (10) together with surface Eqs. (7) and (8), it leads to a set of nonclassical boundary conditions on the top surface of the bulk layer:

11 $$\left. {\sigma_{zz} } \right|_{z = 0} + \tau^{s} \left. {\left( {\frac{{d^{2} u_{z} }}{{dr^{2} }} + \frac{1}{r}\frac{{du_{z} }}{dr}} \right)} \right|_{z = 0} = - p(r)$$

12 $$\left. {\sigma_{zr} } \right|_{z = 0} + \kappa^{s} \left. {\left( {\frac{{d^{2} u_{r} }}{{dr^{2} }} + \frac{1}{r}\frac{{du_{r} }}{dr} - \frac{{u_{r} }}{{r^{2} }}} \right)} \right|_{z = 0} = - q(r)$$

Since the surface part is considered infinitesimally thin and has no bending resistance, the applied couple traction $m(r)$ on the top of the surface-bulk system is transmitted to the bulk layer directly and this yields an additional boundary condition:

13 $$\left. {m_{z\theta } } \right|_{z = 0} = - m(r)$$

The boundary conditions at the bottom of the bulk layer can be readily expressed as

14 $$\left. {u_{r} } \right|_{z = h} = 0$$

15 $$\left. {u_{z} } \right|_{z = h} = 0$$

16 $$\left. {m_{z\theta } } \right|_{z = h} = 0$$

Equations (11)–(16) form a complete set of boundary conditions for the bulk layer accounting for the surface effects.

## Solution procedure

To obtain the closed-form solution of an elastic field within the bulk layer, a method of Hankel transform together with the representation of the displacement field is adopted. In particular, the vertical and radial displacements of the bulk layer undergoing the axisymmetric deformation admit the following representations^66,67^

17 $$u_{r} = - \frac{\partial }{\partial r}\left[ {\ell^{2} \frac{\partial \Psi }{{\partial z}} + \alpha \left\{ {z(1 - \ell^{2} \Delta )\Psi + \Phi } \right\}} \right]$$

18 $$u_{z} = \Psi - \ell^{2} \frac{{\partial^{2} \Psi }}{{\partial z^{2} }} - \alpha \frac{\partial }{\partial z}\left\{ {z(1 - \ell^{2} \Delta )\Psi + \Phi } \right\}$$

where $\alpha = (\lambda + \mu ){/}2(\lambda + 2\mu )$; $\ell = \sqrt {\eta {/}\mu }$ represents the length scale of the bulk material; $\Delta$ is an axisymmetric Laplacian operator; $\Psi = \Psi (r,z)$ and $\Phi = \Phi (r,z)$ are both solutions of the following equation:

19 $$(1 - \ell^{2} \Delta )\Delta \Psi = 0, \, \Delta \Phi = 0$$

The closed-form general solution of Eq. (19) can be readily established by applying Hankel transform method^54,65,68^ and the final results are given by

20 $$\Psi (r,z) = \int\limits_{0}^{\infty } {\left\{ {C_{1} e^{ - \xi (h - z)} + C_{2} e^{ - \xi z} + C_{3} e^{ - \zeta (h - z)/\ell } + C_{4} e^{ - \zeta z/\ell } } \right\}J_{0} (\xi r)\xi d\xi }$$

21 $$\Phi (r,z) = \int\limits_{0}^{\infty } {\left\{ {C_{5} e^{ - \xi (h - z)} + C_{6} e^{ - \xi z} } \right\}J_{0} (\xi r)\xi d\xi }$$

where $J_{m}$ denotes Bessel function of the first kind of order *m*; $\xi \in [0,\infty )$ is a transform parameter; $\zeta = \sqrt {1 + \ell^{2} \xi^{2} }$; and $C_{i} \, (i = 1,2,...,6)$ are unknown coefficients. The general solutions of the displacements $\{ u_{r} ,u_{z} \}$, the rotation $\Omega_{\theta }$, the force stress components $\{ \sigma_{rr} ,\sigma_{\theta \theta } ,\sigma_{zz} ,\sigma_{rz} ,\sigma_{zr} \}$, and the couple stress components $\{ m_{r\theta } ,m_{\theta r} ,m_{z\theta } ,m_{\theta z} \}$ can be obtained upon substitution of Eqs. (20) and (21) into Eqs. (17), (18), (2), and (3). The explicit expressions for the complete elastic field within the bulk layer, in terms of the unknown coefficients $C_{i} \, (i = 1,2,...,6)$, are reported in Supplementary Appendix for the sake of brevity.

By enforcing the boundary conditions given by Eqs. (11)–(16) together with the general solutions for $\{ u_{r} ,u_{z} ,\sigma_{zz} ,\sigma_{zr} ,m_{z\theta } \}$ given in Supplementary Appendix, it yields the following system of linear algebraic equations for determining the unknown coefficients $C_{i} \, (i = 1,2,...,6)$:

22 $${\varvec{A}}(\xi ){\varvec{C}} = {\varvec{F}}(\xi )$$

where $${\varvec{C}} = \{ \begin{array}{*{20}c} {C_{1} } & {C_{2} } & \cdots & {C_{6} } \\ \end{array} \}^{T}$$ and the coefficient matrix ${\varvec{A}}(\xi )$ and the vector ${\varvec{F}}(\xi )$ are given explicitly by

23 $${\varvec{A}}(\xi ) = \left[ {\begin{array}{*{20}c} {(h_{1} + h_{s} )e^{ - \xi h} } & { - h_{1} + h_{s} } & { - \ell \xi (\zeta - \ell \tau^{s} \xi^{2} )e^{ - \zeta h/\ell } } & {\ell \xi (\zeta + \ell \tau^{s} \xi^{2} )} & { - \alpha \xi (1 - \tau^{s} \xi )e^{ - \xi h} } & { - \alpha \xi (1 + \tau^{s} \xi )} \\ {(\alpha - h_{1} - \ell \kappa^{s} \xi^{3} )e^{ - \xi h} } & {\alpha - h_{1} + \ell^{2} \kappa^{s} \xi^{3} } & {\ell \xi^{2} (\ell - \kappa^{s} \zeta )e^{ - \zeta h/\ell } } & {\ell \xi^{2} (\ell + \kappa^{s} \zeta )} & {\alpha \xi (1 - \kappa^{s} \xi )e^{ - \xi h} } & { - \alpha \xi (1 + \kappa^{s} \xi )} \\ {\ell \xi e^{ - \xi h} } & { - \ell \xi } & {\zeta e^{ - \zeta h/\ell } } & { - \zeta } & 0 & 0 \\ {h_{2} } & {h_{3} e^{ - \xi h} } & {\ell \zeta } & { - \ell \zeta e^{ - \zeta h/\ell } } & \alpha & {\alpha e^{ - \xi h} } \\ {1 - \alpha - h_{2} \xi } & {(1 - \alpha + h_{3} \xi )e^{ - \xi h} } & { - \ell^{2} \xi^{2} } & { - \ell^{2} \xi^{2} e^{ - \zeta h/\ell } } & { - \alpha \xi } & {\alpha \xi e^{ - \xi h} } \\ {\ell \xi } & { - \ell \xi e^{ - \xi h} } & \zeta & { - \zeta e^{ - \zeta h/\ell } } & 0 & 0 \\ \end{array} } \right]$$

24 $${\varvec{F}}(\xi ) = \left\{ {\begin{array}{*{20}c} {P(\xi )/\xi } \\ {Q(\xi )/\xi } \\ {M(\xi )/\ell \xi } \\ 0 \\ 0 \\ 0 \\ \end{array} } \right\}$$

with $$h_{1} = 1/2 - \ell^{2} \xi^{2}$$, $$h_{2} = \alpha h + \ell^{2} \xi$$, $$h_{3} = \alpha h - \ell^{2} \xi$$, $$h_{s} = \ell^{2} \tau^{s} \xi^{3} + \alpha \tau^{s} \xi - \tau^{s} \xi$$, and

25 $$P(\xi ) = - \int\limits_{0}^{\infty } {p(r)J_{0} (\xi r)rdr} , \, Q(\xi ) = - \int\limits_{0}^{\infty } {q(r)J_{1} (\xi r)rdr} , \, M(\xi ) = - \int\limits_{0}^{\infty } {m(r)J_{1} (\xi r)rdr}$$

The solution of the system (Eq. 22) for each $\xi \in [0,\infty )$ can be obtained numerically via standard linear solvers. Once $C_{i} \, (i = 1,2,...,6)$ are solved, the elastic field within the bulk layer can be obtained from supplementary Eqs. (A1)–(A12). To evaluate all involved improper integrals, an efficient quadrature rule similar to that employed by Rungamornrat et al.^54^ and Lawongkerd et al.^65^ is adopted.

## Results and discussion

Computed results for certain cases are first compared with existing benchmark solutions to verify both the formulation and solution procedure. The influence of surface and couple stresses on the elastic field within a thin material layer under various surface loads is subsequently investigated. To clearly demonstrate the individual and simultaneous effects on the size-dependent characteristics, results for four different models (i.e., Model-1 with both surface and couple stress effects, Model-2 with only surface effect (i.e., $\ell \to 0$), Model-3 with only couple stress effect (i.e., $\tau^{s} ,\kappa^{s} \to 0$), and Model-4 without surface and couple stress effects (i.e., $\tau^{s} ,\kappa^{s} ,\ell \to 0$)) are reported and compared. For convenience in simulations and presentation of results, following normalized coordinates and parameters $\overline{r} = r{/}\Lambda$, $\overline{z} = z{/}\Lambda$, $\overline{a} = a{/}\Lambda$, $\overline{h} = h{/}\Lambda$, $\overline{\tau }^{s} = \tau^{s} {/}2\mu \Lambda$, and $l_{0} = \ell /\Lambda$ with $\Lambda = \kappa^{s} {/}2\mu$ denoting the length scale of the material surface are introduced.

### Verification

In the numerical study, material parameters reported by Miller and Shenoy^48^ and Shenoy^49^ are employed. In particular, Lamé constants of the bulk material are taken as $\lambda = 58.17 \times 10^{9} {\text{ N/m}}^{2}$ and $\mu = 26.13 \times 10^{9} {\text{ N/m}}^{2}$, whereas surface Lamé constants and the residual surface tension are taken as $\lambda^{s} = 6.8511{\text{ N/m}}$, $\mu^{s} = - 0.376{\text{ N/m}}$, and $\tau^{s} = 1{\text{ N/m}}$, respectively.

**Figure 2:**
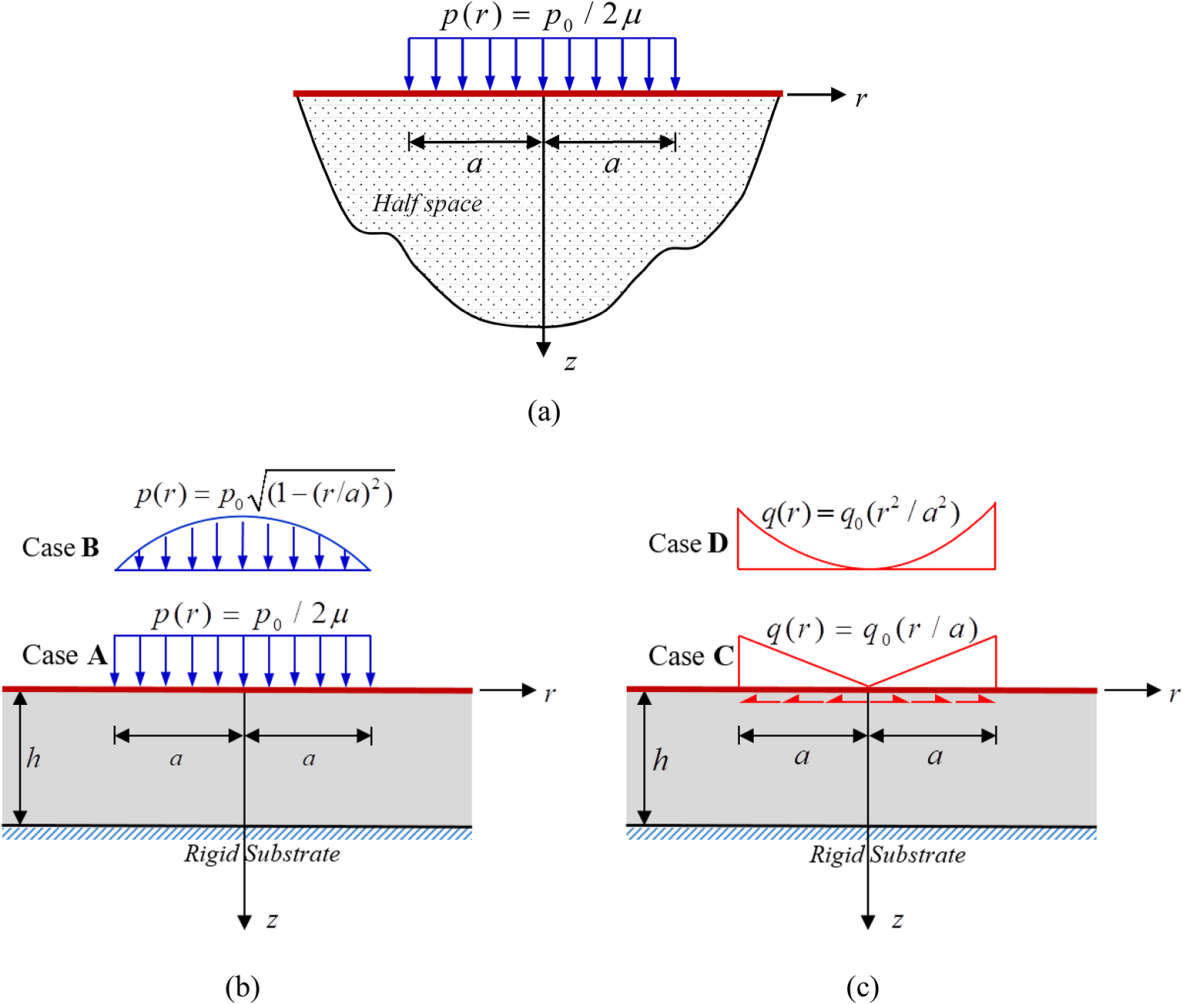
(**a**) Elastic half space under uniformly distributed normal traction; (**b**) elastic layer resting on rigid substrate under uniformly distributed (Case **A**) and Hertzian (Case **B**) normal traction; and (c) elastic layer resting on rigid substrate under linearly distributed (Case **C**) and quadratically distributed (Case **D**) radial shear traction over a circular region of radius $a$.

**Figure 3:**
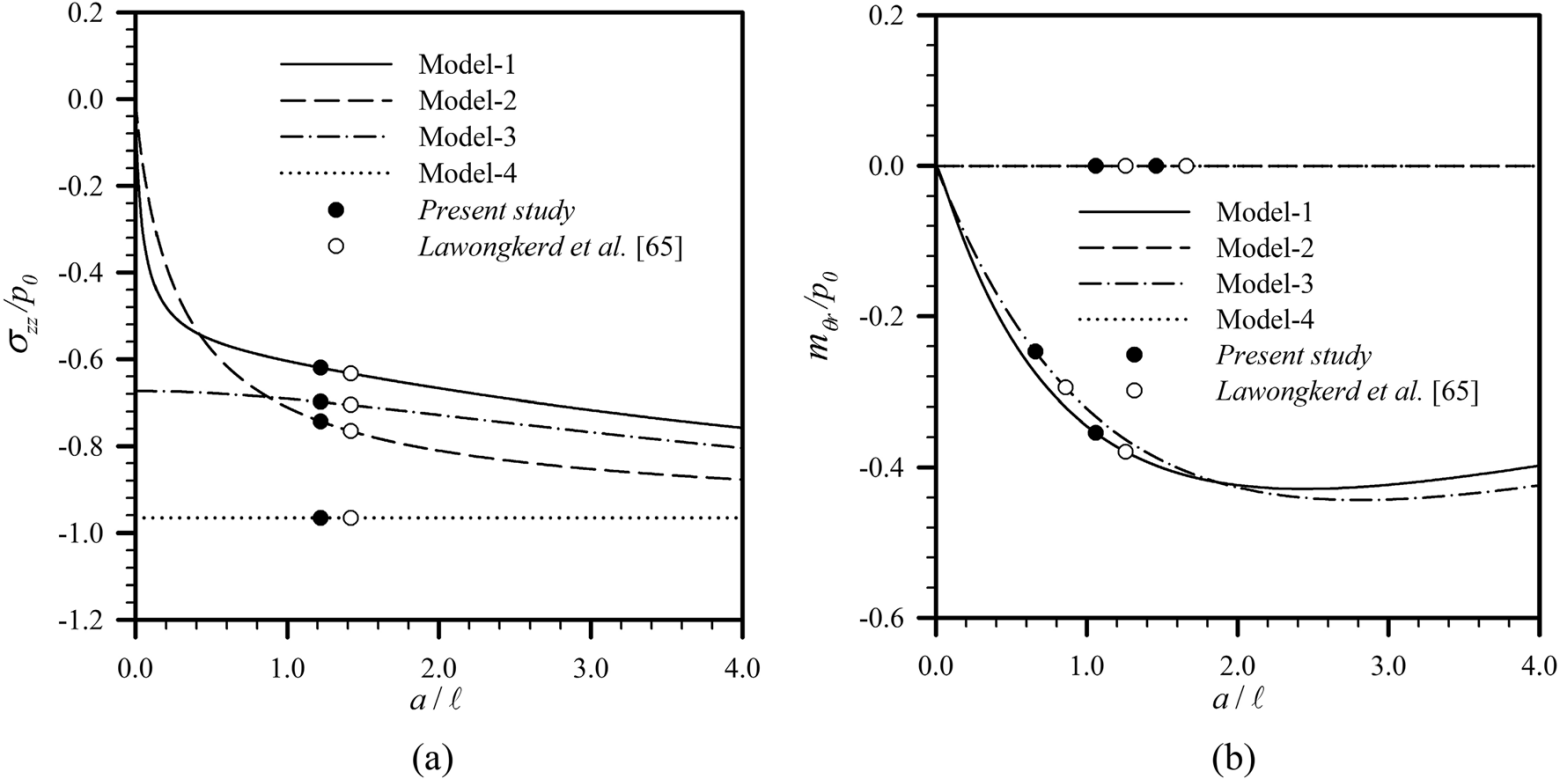
Variations of (**a**) normalized vertical stress and (**b**) normalized couple stress of an infinite elastic layer under uniformly distributed normal traction for $z{/}a = 0.25$, $r/a = 0.5$, and $l_{0} = 1$.

Consider first an elastic half space subjected to a uniformly distributed normal traction $p_{0}$ over a circular region of radius $a$ as illustrated in Fig. 2a. To simulate the half-space medium within the current setting, the thickness of the layer is taken to be sufficiently large in comparison with $a$ and the ratio $h{/}a = 1000$ is considered in the analysis. Results for the force stress component $\sigma_{zz}$ and the couple stress component $m_{\theta r}$ versus the ratio $a{/}\ell$ are compared with those reported by Lawongkerd et al.^65^ in Fig. 3 for $z{/}a = 0.25$, $r/a = 0.5$, and $l_{0} = 1$. It is seen that the computed results are in excellent agreement with the benchmark solutions for all four models.

**Figure 4:**
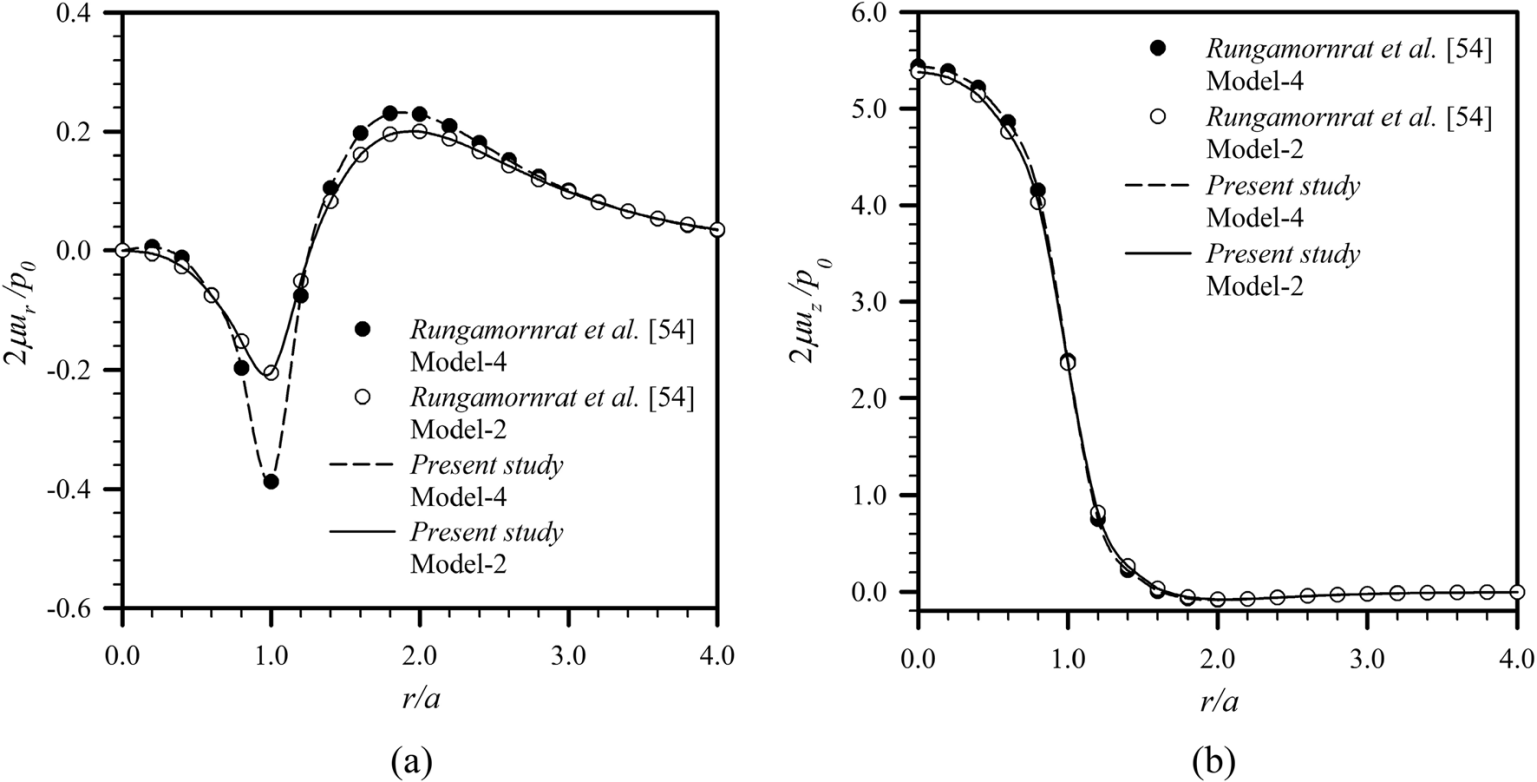
Normalized displacement profiles of an infinite elastic layer under uniformly distributed normal traction: (**a**) radial displacement and (**b**) vertical displacement.

**Figure 5:**
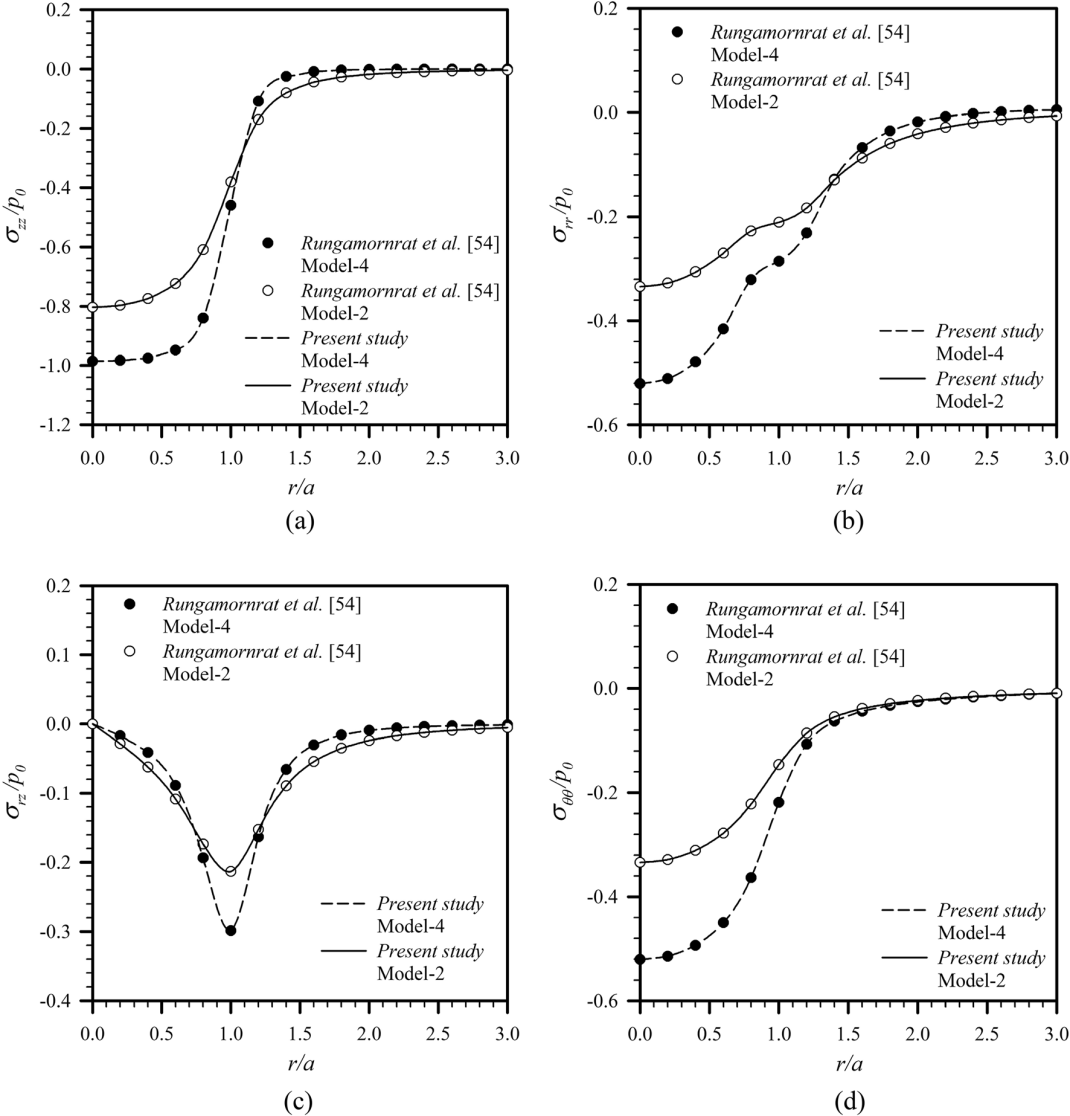
Normalized stress profiles of an infinite elastic layer under uniformly distributed normal traction: (**a**) vertical stress, (**b**) radial stress, (**c**) shear stress, and (**d**) hoop stress.

Another verification is carried out for an elastic layer under a uniformly distributed normal traction $p_{0}$ acting on a circular region of radius $a$ shown in Fig. 2b for the load Case **A**. Results for this particular problem were reported by Rungamornrat et al.^54^ for the classical case and the case with only surface stress effect. To simulate these two special cases, the parameters $\tau^{s} ,\kappa^{s} ,\ell$ and $\ell$ are taken to be sufficiently small for each scenario. The computed surface displacements (i.e., $\overline{z} = 0$) are reported in Fig. 4 for $\overline{a} = 10$ and $\overline{h} = 10$ and the stress components at the normalized depth $\overline{z} = 0.25$ are shown in Fig. 5 for $\overline{a} = 1$ and $\overline{h} = 10$. The good agreement between the two sets of results additionally confirms the validity of the proposed scheme and derived solutions.

### Influence of surface and couple stresses

In this section, results from a parametric study are reported to demonstrate the role of both surface and couple stresses on the predicted response and size-dependent behavior of a substrate coated by a thin coating layer under surface loads. In particular, the load transferring characteristics from the coating surface to the substrate and the influence of the coating-layer thickness are of primary interest. To also explore the influence of applied loads and their distribution, a coated system subjected to four representative surface loads acting on a circular region of radius $a$ shown in Fig. 2b,c (i.e., Case **A** for a uniformly distributed normal traction $p(r) = p_{0}$, Case **B** for the Hertzian normal traction $p(r) = p_{0} \sqrt {1 - (r{/}a)^{2} }$, Case **C** for a linearly distributed radial shear traction $q(r) = q_{0} r/a$, and Case **D** for a quadratically distributed radial shear traction $q(r) = q_{0} (r{/}a)^{2}$) are considered. In simulations, the following material parameters $E = 76{\text{ GPa}}$, $\nu = 0.3$, $\kappa^{s} = 1.22{\text{ N/m}}$, and $\tau^{s} = 0.89{\text{ N/m}}$^48,49^ are utilized unless stated otherwise. Note in addition that only the case of comparable surface and couple stress effects is investigated and, to simulate such scenario, the two material length scales $\ell ,\Lambda$ are taken as $l_{0} = 1$. The full discussion on the two size effects for a wide range of the ratio $\ell {/}\Lambda$ can be found in the work of Le et al.^63^ and Lawongkerd et al.^65^.

**Figure 6:**
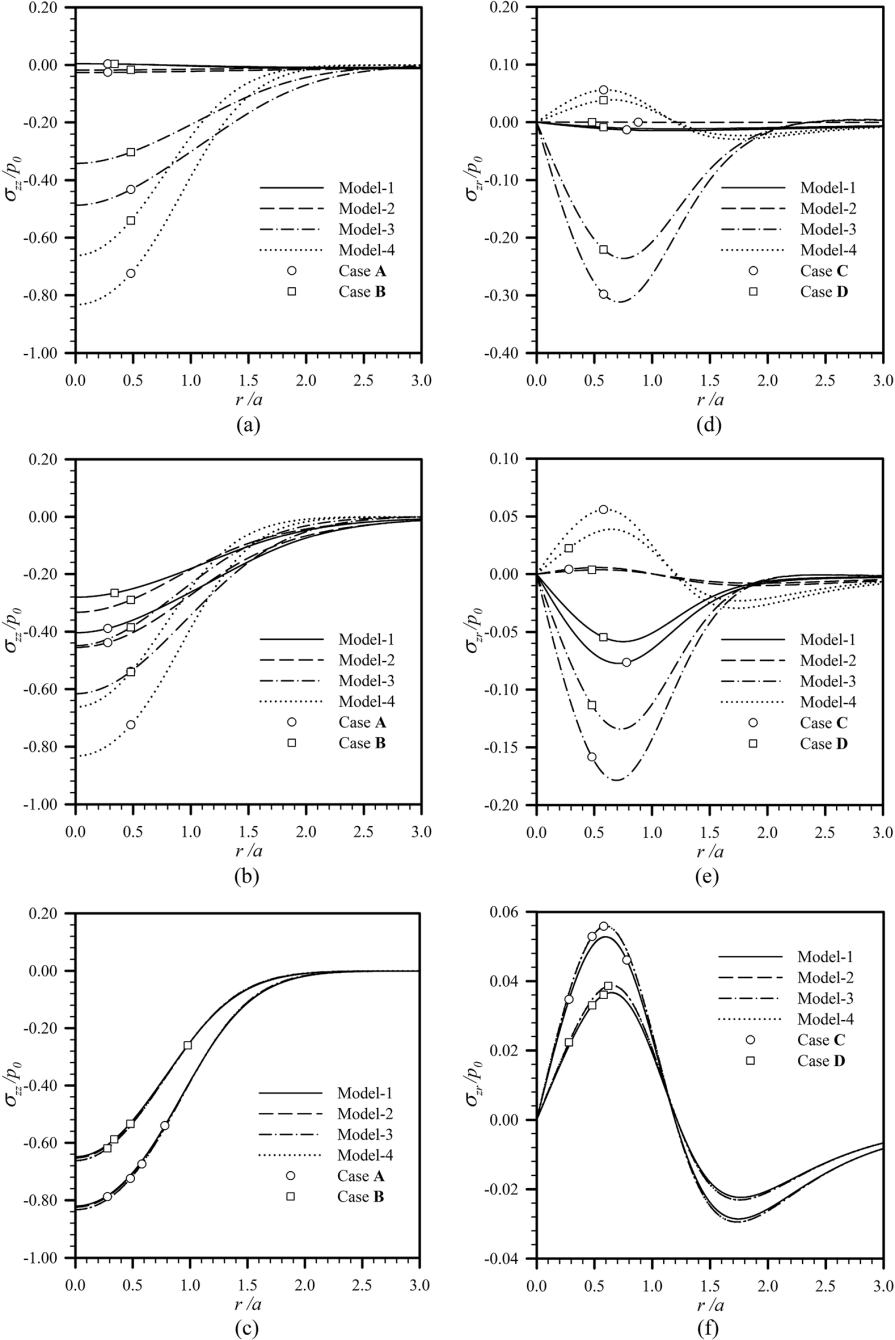
Profiles of normalized vertical and shear stresses in the radial direction at the bottom of coating layer for $h{/}a = 1$ and $l_{0} = 1$: (**a,d**) $a{/}\ell = 0.01$, (**b,e**) $a{/}\ell = 1$, and (**c,f**) $a{/}\ell = 100$.

To demonstrate the intensity of load transferring to the coated substrate, the vertical stress $\sigma_{zz}$ for the load Case **A** and load Case **B** and the shear stress $\sigma_{zr}$ for the load Case **C** and load Case **D** are reported in Fig. 6 for $z{/}a = 1$, $h{/}a = 1$, and $a{/}\ell \in \{ 0.01,1,100\}$. Three values of the ratio $a{/}\ell$ are considered to represent cases when the size of a loading region (representing the external length scale) is much less than, comparable to, and much larger than the two material length scales. Note that both vertical and shear stresses are normalized by the maximum intensity of the applied surface loads to clearly observe the role of the coating layer in the reduction of the transferring stresses to the substrate. For the first two load cases (i.e., load Case **A** and load Case **B**), the normalized vertical stress attains the maximum magnitude at the center of the loading region and monotonically decays to zero as $r{/}a$ increases for all models and values of $a{/}\ell$ (see Fig. 6a–c). As the size of the loading region becomes comparable to both bulk and surface material length scales, transferring vertical stresses to the substrate are clearly different for all four models (see Fig. 6b). Such finding confirms the important role of both surface and couple stresses when $a$ fall within the range of $\ell ,\Lambda$. Clearly, the Model-2 and Model-3 cannot be used as the replacement of the Model-1. In addition, the presence of surface and couple stress effects clearly reduce the maximum transferring stress to the substrate in comparison with the classical case; in particular, the Model-1 yields the least value of the maximum transferring stress. When $a$ is much less than $\ell ,\Lambda$ (see Fig. 6a), the Model-2 and Model-3 still predict responses differently from the classical case, but the effect of surface stresses is more pronounced than that of the couple stresses. The transferring vertical stresses obtained from the Model-1 and Model-2 are comparable but very different from those from the Model-3 and Model-4. These results suggest that the Model-2 can be used in lieu of the Model-1 to simplify the calculations when $a \ll \ell \sim \Lambda$. When $a$ is much larger than $\ell ,\Lambda$ (see Fig. 6c), the surface and couple stresses play an insignificant role in the predicted response; in particular, results from the Model-1, Model-2, and Model-3 are almost identical to the classical solutions. For this range of external and material length scales, the Model-4 is considered sufficient for simulating the response of interest. It is worth noting that changing the distribution of applied normal tractions does not alter the response characteristics except for the difference in magnitude resulting from the difference in the traction resultant. For the load Case **C** and load Case **D**, the magnitude of the normalized shear stress $\sigma_{zr} {/}q_{0}$ transferring to the substrate increases from zero at the center of the loading region to its maximum at $r{/}a \in [0.5,1]$ and then decays asymptotically to zero as $r$ increases (see Fig. 6d–f). It is worth pointing out that for these loading conditions, the role of the surface stresses on the maximum transferring shear stress is opposite to that of the couple stresses. Specifically, the surface stresses (the Model-2) tend to lower the maximum transferring shear stress from the classical case while the couple stresses clearly boost such maximum and also switch the direction of the shear stress. By comparing results for three different values of $a{/}\ell$ and two different distributions of the applied shear loads, a similar conclusion to the load Case **A** and load Case **B** can be drawn. In particular, as the size of the loading region reduces to be comparable to (or much less than) the two length scales $\ell ,\Lambda$, the Model-1 (or the Model-1 and Model-2) must be used to capture the size effects. Note also that the presence of both surface and couple stresses can either reduce (as $a \ll \ell \sim \Lambda$) or boost (as $a \sim \ell \sim \Lambda$) the maximum transferring shear stress to the substrate from the classical case.

**Figure 7:**
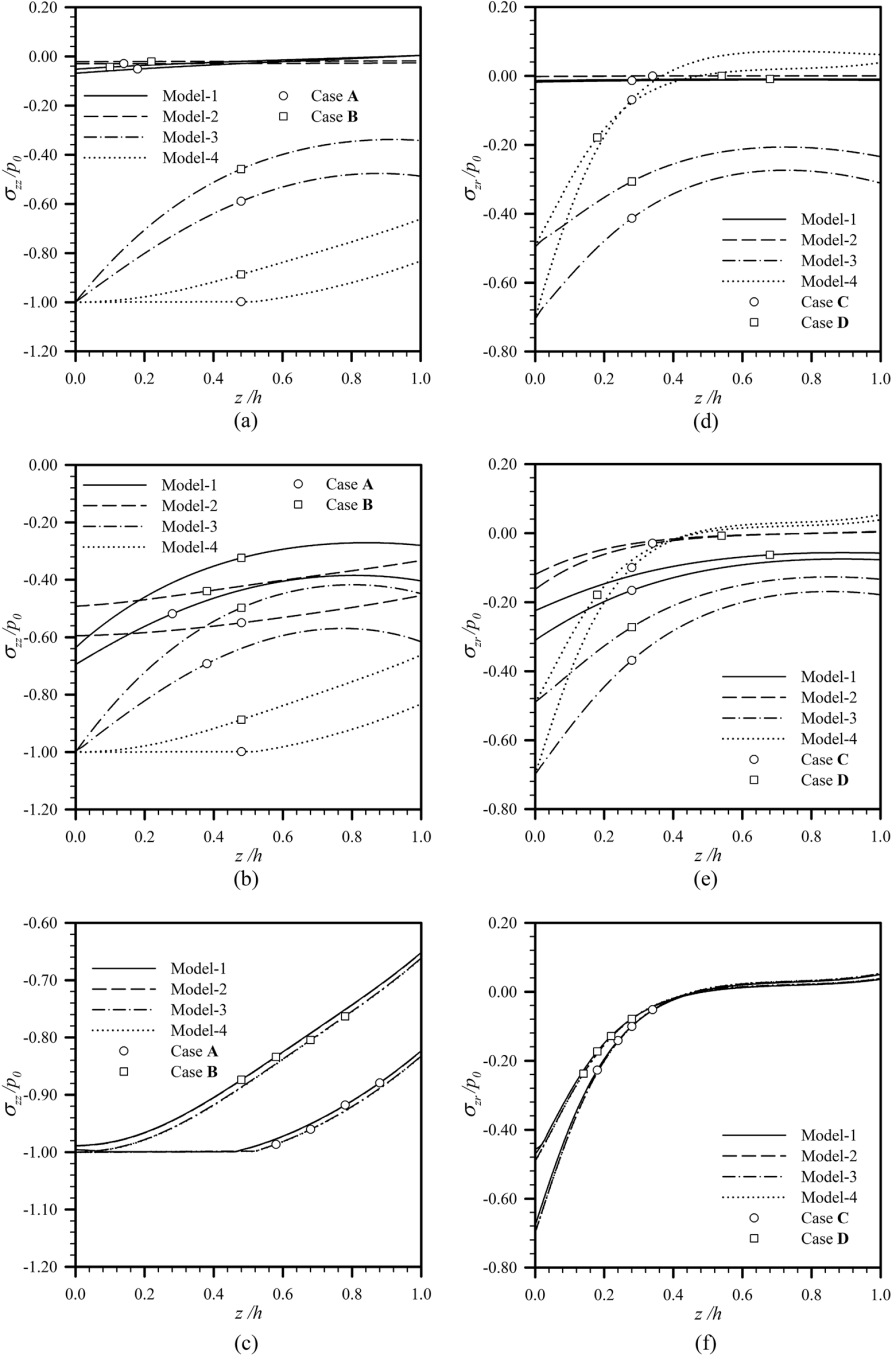
Through-the-thickness profiles of normalized vertical stress at $r{/}a = 0$ and shear stress at $r{/}a = 0.7$ for $h{/}a = 1$ and $l_{0} = 1$: (**a,d**) $a{/}\ell = 0.01$, (**b,e**) $a{/}\ell = 1$, and (**c,f**) $a{/}\ell = 100$.

Through-the-thickness profiles of the vertical stress $\sigma_{zz}$ at $r{/}a = 0$ for the load Case **A** and load Case **B** and the shear stress $\sigma_{zr}$ at $r{/}a = 0.7$ for the load Case **C** and load Case **D** are also reported in Fig. 7 for $h/a = 1$ and $a{/}\ell \in \{ 0.01,1,100\}$. The specific values of $r{/}a$ used to collect those results are associated with the location where the transferring stress to the substrate attains (for the load Case **A** and load Case **B**) or approximately attains (for the load Case **C** and load Case **D**) its maximum. It is evident from Fig. 7a–c that the vertical stress predicted by the Model-1, Model-2 and Model-3 decreases faster than those in the classical case as the depth $z$ increases. Nevertheless, for load Case **C** and load Case **D** (see Fig. 7d–f), the Model-2 tends to boost the decay of the shear stress across the thickness of the coating layer from the classical case, but the Model-3 seems to lower such decay. The Model-1 accounting for both effects can either lower (see Fig. 7e) or boost (see Fig. 7d) the decay depending on the ratio $a{/}\ell$. For all load cases considered, the through-the-thickness profiles of both vertical and radial shear stresses are strongly dependent on both surface and couple stress effects when $a$ is comparable to (see Fig. 7b,e) or much less than (see Fig. 7a,d) $\ell ,\Lambda$ and for the latter case, the surface effect is found to be more pronounced. Note in addition that changing the distribution of applied surface loads does not alter the trend of the predicted response.

**Figure 8:**
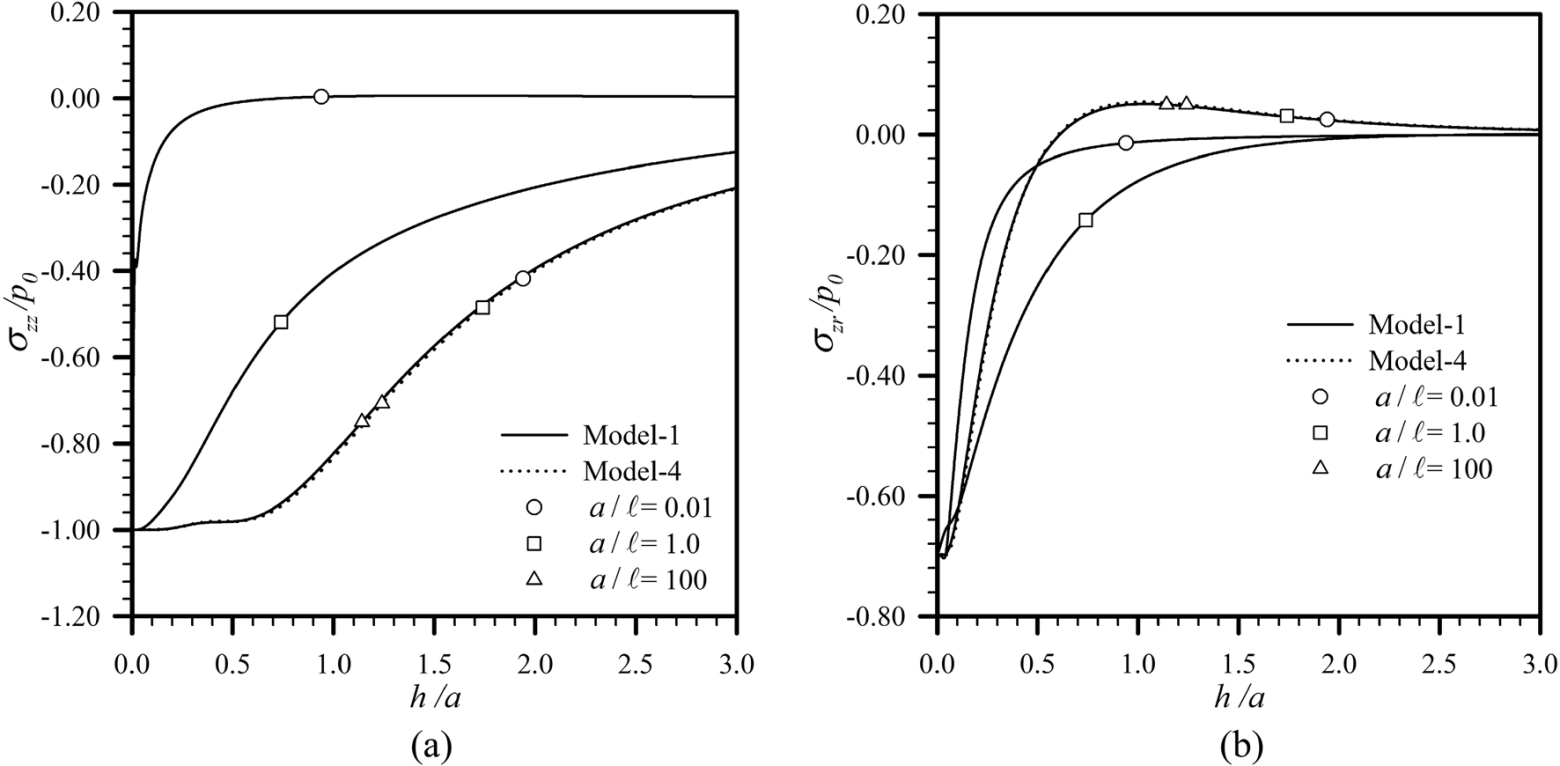
Normalized (**a**) maximum transferring vertical stress for load Case **A** and (**b**) transferring shear stresses at $r{/}a = 0.7$ for load Case **C** versus normalized thickness of coating layer. Results are reported for $l_{0} = 1$ and $a{/}\ell \in \{ 0.01,1,100\}$.

To further illustrate the influence of the coating-layer thickness on the reduction of the transferring stress on the substrate when both surface and couple stress effects are present, the transferring vertical and shear stresses for the case of applied normal and radial shear stresses, obtained from the Model-1, are reported as a function of the normalized thickness $h{/}a$ in Fig. 8 for $a{/}\ell \in \{ 0.01,1,100\}$. Since the role of surface and couple stress effects for different surface load distributions are similar, the load Case **A** and load Case **C** are chosen as representative load cases for applied normal and radial shear tractions, respectively. For the load Case **A**, the transferring vertical stress $\sigma_{zz}$ is reported at $r{/}a = 0$ where it attains the maximum (see Fig. 6a–c). For this loading case, the increase in the thickness of the coating layer can significantly lower the maximum transferring vertical stress to the substrate for both the classical model and Model-1. However, the presence of both couple and surface stresses renders such reduction more pronounced when the size of the loading region either falls within the range of or is much smaller than the material length scales $\ell ,\Lambda$ (see Fig. 8a). For the latter case (as $a \ll \ell \sim \Lambda$), the surface effect is the key responsible for such substantial reduction from the classical case. For the load Case **C**, it is chosen, for convenience, to report the transferring radial shear stress $\sigma_{zr}$ at $r{/}a = 0.7$ since the exact location of the maximum $r{/}a$ varies from 0.5 to 1 (see Fig. 6d–f). It is seen from this set of results that while the transferring shear stress to the substrate decreases monotonically and asymptotically to zero as the coating-layer thickness increases when both surface and couple stresses are taken into account, but the presence of such effects can either enhance (as $a \sim \ell \sim \Lambda$) or reduce ($a \ll \ell \sim \Lambda$) the transferring shear stress from the classical case. In addition, the switch of the direction of the transferring shear stress from that of the applied shear traction for a sufficiently large $h{/}a$, as observed for the classical case, disappears when both surface and couple stress effects are significant.

**Figure 9:**
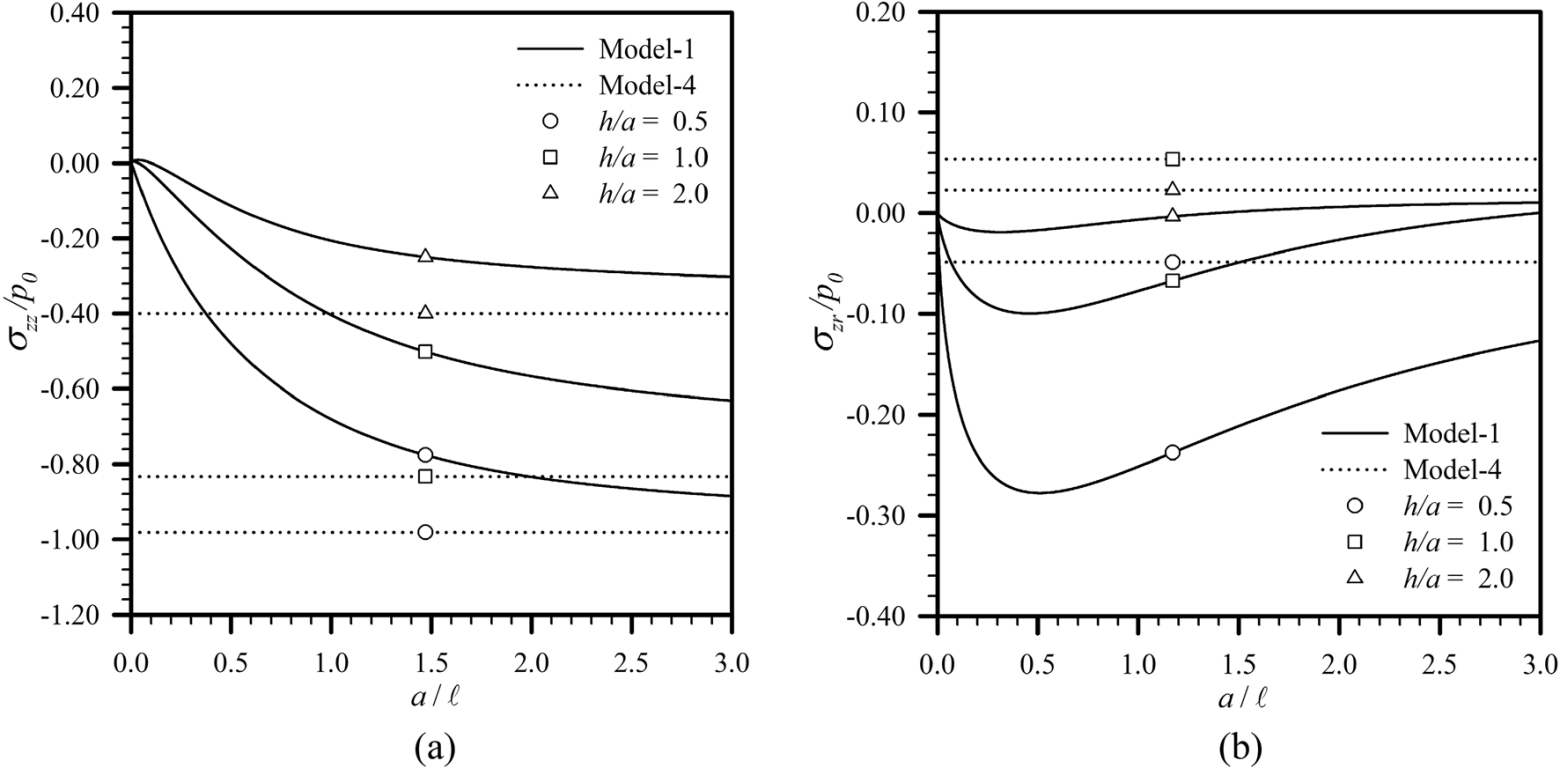
Normalized (**a**) maximum transferring vertical stress for load Case **A** and (**b**) transferring shear stresses at $r{/}a = 0.7$ for load Case **C** versus the ratio $a{/}\ell$. Results are reported for $l_{0} = 1$ and $h{/}a \in \{ 0.5,1,2\}$.

Finally, the size dependent characteristics of the predicted transferring stresses to the substrate are also investigated. To clearly illustrate such behavior, the maximum transferring vertical stress for the load Case **A** and the transferring shear stress at $r{/}a = 0.7$ for the load Case **C** are reported as a function of $a{/}\ell$ in Fig. 9 for $h{/}a \in \{ 0.5,1,2\}$. It is seen that for any given aspect ratio $h{/}a$, the normalized transferring stresses to the substrate obtained from the Model-1 are strongly size dependent or, equivalently, depend on the length scale ratio $a{/}\ell$. As $a$ decreases to be comparable to or less than $\ell$, the maximum transferring vertical stress for the load Case **A** drops quite rapidly and monotonically from the value predicted by the classical model. The different behavior is observed for the load Case **C**. The variation of the transferring shear stress over a wide range of the ratio $a{/}\ell$ is not monotone; in particular, for $a$ comparable to or larger than $\ell ,\Lambda$, the predicted transferring shear stress from the Model-1 is higher than the classical solution while the reverse trend can be concluded when $a$ is much less than $\ell ,\Lambda$. For both loading cases, the size dependency decays to be insignificant as the size of the loading region $a$ is much larger than the material length scales $\ell ,\Lambda$, and the Model-4 is therefore sufficient for the simulations.

## Conclusion

The analytical solution of an elastic field of a thin material layer coating a rigid substrate and excited by axisymmetrically distributed surface loads have been derived. Such established results are considered novel in that both surface free energy and material microstructures, which are recognized to be responsible for the size effects in small-scale objects, are taken into account simultaneously to model a medium of finite thickness. This allows the direct application to simulate the mechanical response of components coated by a very thin material layer. A continuum-based model integrating the couple stress elasticity theory for tackling the inherent microstructural effect and Gurtin–Murdoch surface elasticity theory for capturing the surface effect has been formulated and solved by an analytical scheme based on Hankel transform and the displacement representation. Obtained results are explicit in an integral form, highly accurate as useful benchmark solutions, and an essential basis for the development of solution schemes to tackle surface contact problems.

Results from an extensive numerical study have revealed that the surface and couple stresses significantly affect both the characteristics and maximum value of load transferring to the coated substrate in comparison with the classical case when the size of the loading region is comparable to or much smaller than the material length scales. For a coated system under normal tractions, the presence of surface and couple stresses can significantly boost the reduction of the transferring vertical stress to the substrate especially when the size of the loading region is much less than the length scale of the bulk and surface materials. A different trend has been observed in the case of applied shear loads. The transferring shear stress to the substrate predicted by the model integrating both surface and couple stress effects can be either lower or higher than the classical solution depending on the ratio between the size of the loading region and the material length scales. This results directly from that the surface effect lowers the transferring shear stress but the couple stress effect causes the reverse trend. In addition, as the size of the loading region become much smaller than the two material length scales, the surface effect is much more pronounced than the couple stress effect.

## Supplementary Information

Supplementary Information.

## Data Availability

The datasets generated and/or analyzed during the current study are not publicly available due to that the data also forms part of an ongoing study, but are available from the corresponding author on reasonable request.
