# Supplementary material for: Elastic solution of surface loaded layer with couple and surface stress effects
Source: Sci Rep. 2023 Jan 19;13:1033. doi: 10.1038/s41598-023-27705-1 (PMC9852297; doi:10.1038/s41598-023-27705-1)
Supplement: Supplementary file 1 — Supplementary Information. [file 41598_2023_27705_MOESM1_ESM.doc]

# appendix

By substituting the general solution of and into Eqs. (20)-(21) into Eqs. (17)-(18), (2), and (3), it finally yields the general solution of the displacement, rotation, force stresses, and couple stresses:

(A1)

(A2)

(A3)

(A4)

(A5)

(A6)

(A7)

(A8)

(A9)

(A10)

(A11)

(A12)

where $\beta=\eta'/\eta$.
